# EAST MEETS WEST: BUILDING DEMENTIA-FRIENDLY COMMUNITIES: REFLECTIONS ON GLOBAL EXPERIENCES

**DOI:** 10.1093/geroni/igz038.1664

**Published:** 2019-11-08

**Authors:** Fei Sun, Nancy Hooyman

**Affiliations:** 1 Michigan State University, East Lansing, Michigan, United States; 2 University of Washington, Seattle, United States

## Abstract

The emergence and rapid development of dementia friendly initiatives (DFIs) represents growing global awareness of needs of persons living with dementia (PWD). Policy and practice efforts across regions and countries made to make physical and social environments more friendly, inclusive, and capable for PWD vary. When designing, implementing and evaluating DFIs, countries at different socioeconomic development stages need to set priorities based upon their local needs and cultural norms. This East Meets West symposium aims to understand the variability and progress of the DFI in the global context, highlighting experiences from two countries: U.S.A. and China, where about one third of the world’s total estimated 47 million PWD live. The first study from Shanghai emphasizes efforts by local health care professionals to promote dementia screening and improve diagnosis, with the ultimate goal to improve dementia literacy and build a dementia friendly city. The second paper describes a community-based participatory approach to develop dementia friendly communities in Beijing, highlighting the significance of non-governmental and governmental collaboration. The DFI in Florida underscores the partnership among multiple sectors with an emphasis of the involvement of PWD, while the DFI in rural Michigan stresses care system coordination. The last paper based upon an online survey of DFI stakeholders from six WHO regions echoes successful features of DFIs (i.e., involvement of PWD, multi-sectorial partnership) identified in Chinese and U.S. examples, and identifies variations in strategies used to modify physical and social environments across countries. Invited panelists from different sectors will share comments in the end.

